# Variation in pH gradients and *FLO11* expression in mat biofilms from environmental isolates of the yeast *Saccharomyces cerevisiae*

**DOI:** 10.1002/mbo3.1277

**Published:** 2022-03-25

**Authors:** Amy L. Forehand, Dulguun Myagmarsuren, Ziyan Chen, Helen A. Murphy

**Affiliations:** ^1^ Department of Biology William & Mary Williamsburg Virginia USA; ^2^ Present address: Amy L. Forehand, Department of Biology University of Virginia Charlottesville Virginia USA; ^3^ Present address: Ziyan Chen, School of Medicine University of Virginia Charlottesville Virginia USA

**Keywords:** adhesin, biofilm, *FLO11*, multicellular, yeast mat

## Abstract

*Saccharomyces cerevisiae* produces a multicellular phenotype, known as a mat, on a semi‐solid medium. This biofilm phenotype was first described in the lab strain Σ1278b and has been analyzed mostly in this same background. Yeast cells form a mat by spreading across the medium and adhering to each other and the surface, in part through the variegated expression of the cell adhesion, *FLO*11. This process creates a characteristic floral pattern and generates pH and glucose gradients outward from the center of the mat. Mats are encapsulated in a liquid which may aid in surface spreading and diffusion. Here, we examine thirteen environmental isolates that vary visually in the phenotype. We predicted that mat properties were universal and increased morphological complexity would be associated with more extreme trait values. Our results showed that pH varied significantly among strains, but was not correlated to mat complexity. Only two isolates generated significant liquid boundaries and neither produced visually complex mats. In five isolates, we tracked the initiation of *FLO11* using green fluorescent protein (GFP) under the control of the endogenous promoter. Strains varied in when and how much GFP was detected, with increased signal associated with increased morphological complexity. Generally, the signal was strongest in the center of the mat and absent at the expanding edge. Our results show that traits discovered in one background vary and exist independently of mat complexity in natural isolates. The environment may favor different sets of traits, which could have implications for how this yeast adapts to its many ecological niches.

## Graphical abstract

Traits previously associated with yeast mat biofilms were quantified in environmental yeast isolates and found to vary significantly. The expression of a major cellular adhesin, *FLO11*, was associated with mat phenotypic complexity. Factors shown to regulate its expression, such as glucose and pH, varied independently of mat complexity.

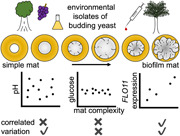

## 1 BACKGROUND

Multicellular phenotypes are found throughout microbial species and can provide fitness benefits ranging from cooperative foraging to protection from environmental stressors (West et al., 2007). The budding yeast *Saccharomyces cerevisiae* exhibits numerous multicellular phenotypes, including flors, flocs, pseudohyphal and invasive growth, and biofilms on solid and semi‐solid surfaces (Verstrepen & Klis, 2006). Biofilms that form on viscous, low agar medium are known as mats (Reynolds & Fink, 2001). The inducing medium is thought to mimic rotting fruit (Pitoniak et al., 2009), a habitat which the budding yeast is famous for exploiting (Greig & Leu, 2009), suggesting the mat phenotype may have relevance to yeast ecology. Most of the research describing mats has focused on a genetically tractable, biofilm‐forming lab strain, Σ1278b; the phenotype is briefly described below.

**Figure 1:**
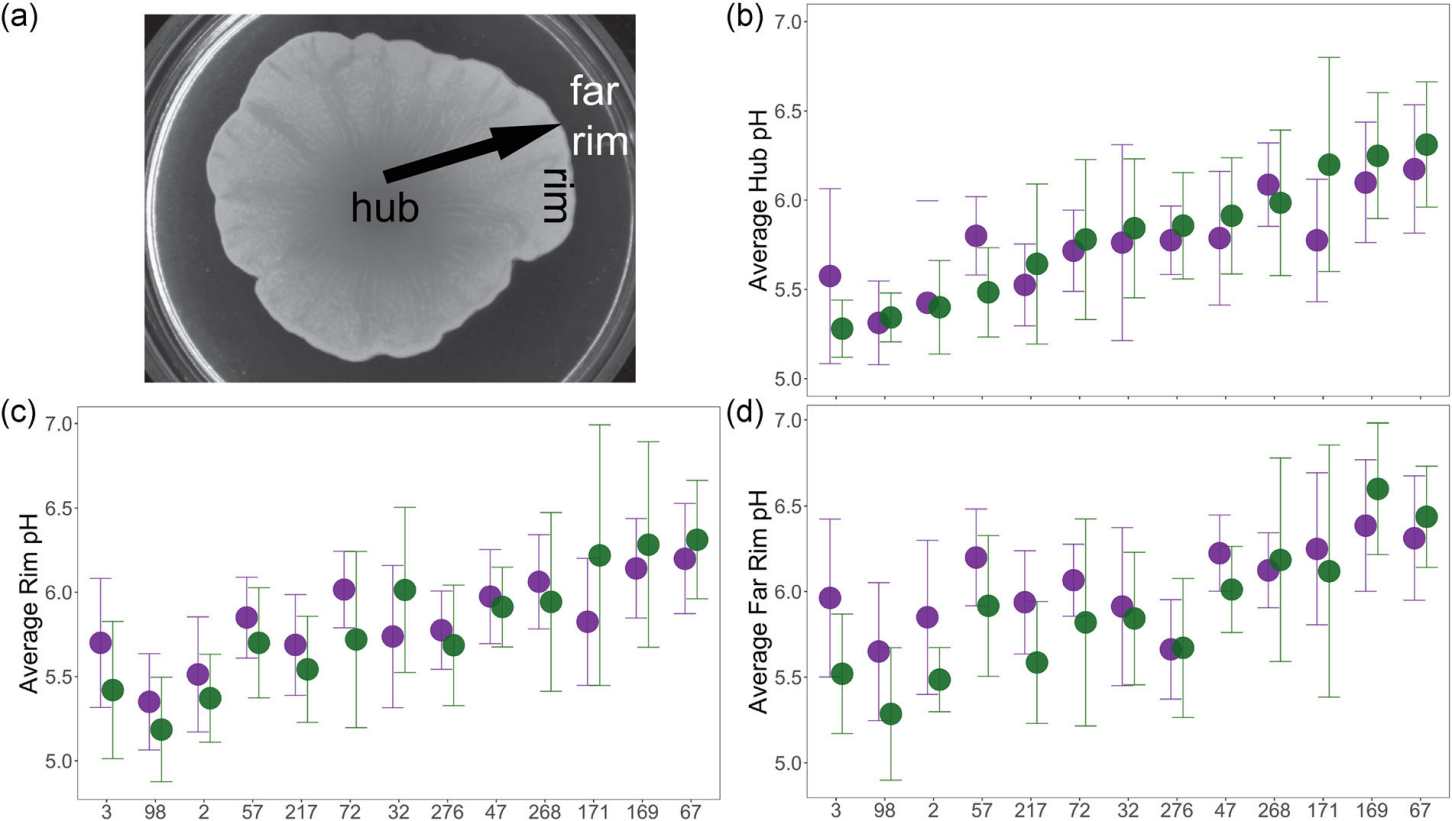
Mat structure and pH measurements. (a) Photo of a yeast mat identifying the hub, rim, and far rim, and the direction of the previously described pH and glucose gradients (Reynolds et al., 2008). (b‒d) Hub, rim, and far rim pH (±2 S.E.M.) in a panel of strains averaged over eight replicates (two mats in each of four assays). Data are presented in order of lowest to highest hub pH at 10 days; 5‐day measurements in green and 10‐day measurements in purple. The *x*‐axis lists the HMY strain number

During growth, mat biofilms spread over a large area, monopolizing access to nutrients and forming a floral pattern (Figure 1a). Variegated expression of the cell adhesin, *FLO11*, is required for mat expansion (Reynolds & Fink, 2001). When cultured on a low agar medium, strains with wild‐type expression generate higher biomass than strains with either constitutive or no expression (Regenberg et al., 2016). Flo11p enables yeast to adhere to surfaces, as well as to each other through homotypic interactions between neighboring cells (Barua et al., 2016; Brückner et al., 2020; Douglas et al., 2007; Dranginis et al., 2007; Reynolds & Fink, 2001). These adherence properties, along with variegated expression of the adhesin, likely contribute to the ability of the yeast to expand during mat formation (Regenberg et al., 2016).

*FLO11* has one of the largest promoters in the yeast genome. It is a hub of regulatory activity: multiple conserved signaling pathways converge (Rupp et al., 1999), transcription of two noncoding RNAs acts as an expression toggle (Bumgarner et al., 2009, 2012), and chromatin remodeling leads to epigenetic silencing (Barrales et al., 2012; Halme et al., 2004). The multiple layers of regulation suggest the importance of precise *FLO11* expression and may lead to fine‐tuned heterogeneity at the population level (Octavio et al., 2009). Indeed, part of the extensive promoter region appears to be under balancing selection (Oppler et al., 2019), perhaps related to adjusting population‐level expression in the environment.

Biofilm mats have a characteristic appearance with a filigreed surface, particularly in the center, as well as channels and spokes toward the outer edges (Reynolds, 2018). Despite *FLO11* expression throughout the mat, the central parts adhere to the surface of the agar, while the outer edge (the rim) does not, suggesting a form of differentiation within the biofilm (Reynolds et al., 2008). Mats also produce a liquid outer layer containing the shed mucins Flo11p and Msb2p, which may function in nutrient availability, communication, and as a lubricant in surface spreading (Karunanithi et al., 2010; Pitoniak et al., 2009).

Mat formation generates both pH and glucose gradients across the agar medium (Reynolds et al., 2008). The pH gradient, which has the lowest pH in the center and the highest at the far rim, may influence the expression (Cullen & Sprague, 2012) and function of Flo11 (Kraushaar et al., 2015). The pH gradient could, in part, explain some apparent differentiation within the mat: an increase in the pH of the medium leads to a decrease in adherence and channeling, while a decrease in the pH medium has the opposite effect (Reynolds et al., 2008). The glucose gradient shows almost no glucose in the center of the mat and increases toward the far rim. The existence of glucose at the rim of the mats suggests that the expanding community is not exhausting all the locally available nutrients as it grows and may be a mechanism by which biofilm‐forming strains outcompete nonbiofilm forming strains (Regenberg et al., 2016). The glucose gradient may also affect *FLO11* expression, as low glucose availability is a positive regulator of the gene (Kuchin et al., 2002).

Although the mat phenotype was described in the lab strain Σ1278b, screens of environment yeast isolates from a variety of habitats have shown that ~10%–25% form mat biofilms (Hope & Dunham, 2014; Oppler et al., 2019; Regenberg et al., 2016). The identification of the phenotype has been based purely on the characteristic appearance described in Σ1278b. However, the colony phenotypes of environmental isolates on low agar exist on a continuum of size and cabling complexity, with haploid strains exhibiting more complexity than their diploid parents (Hope & Dunham, 2014). Within the range of possible phenotypes, it is not always obvious whether a strain is forming a biofilm; therefore, in this article, we refer to all colonies forming on low‐agar surfaces as mats, and mats with visual complexity—large surfaces, floral‐like patterning, and characteristic cabling— as biofilm mats.

It is unknown whether the spatial arrangement of *FLO11* expression, pH and glucose gradients, and the existence of an encapsulating liquid are manifest in environmental isolates. The genetic basis of mat formation is complex with at least 600 genes influencing its expression (Chow et al., 2019; Ryan et al., 2012); natural genetic variation could affect any of these characteristics (Gasch et al., 2016). These traits may be hallmarks of mat biofilm formation and present in all biofilm mats, albeit to varying degrees, or the characteristics may vary independently of one another.

Given the potential relevance of mat biofilms to yeast ecology and how little is known about the phenotype in environmental isolates, we sought to understand the distribution of traits related to mat formation. We assembled environmental isolates from existing collections that varied visually in their mat phenotypes (i.e., size and amount of cabling) and measured the size of the liquid boundary and pH and glucose gradients. In a subset of the isolates, we tracked the initiation of *FLO11* expression over time. We hypothesized that as mat size and cabling complexity increased, pH and glucose gradients would become stronger, the size of the liquid boundary layer would increase, and *FLO11* expression would increase in intensity over a larger spatial area. However, we found that the most visually extreme mat biofilms did not have the most extreme trait values. Furthermore, we found that some medium‐sized, less morphologically complex mats have some of the most pronounced characteristics previously associated with mat biofilms.

## 2 MATERIALS AND METHODS

### 2.1 Strains

Thirteen strains were chosen to sample mat diversity, as well as *FLO11* coding and regulatory diversity (Oppler et al., 2019); the majority were from the 100‐Genomes (Strope et al., 2015) or Sanger (Liti et al., 2009) collections (Table 1). Most were diploid progeny from a single spore, except HMY2, HMY3, and HMY217 which were diploids in the original highly‐heterozygous state (Magwene et al., 2011). We included Σ1278b in our panel, which we obtained from the 100‐Genomes collection. This version of the strain has decreased biofilm formation due to accumulated mutations, including in *RIM15* (Granek & Magwene, 2010); our data are therefore not directly comparable to previously published reports of its mat characteristics.

**Table 1:** Strains

Strain	Strain collection	Regulatory allele	Background	Origin	Location	Strain *prFLO11‐GFP‐KanMX*
HMY2		A (+)	YJM224	Distillery	N/A	HMY568
HMY3		A	YJM311	Clinical	CA, USA	HMY518
HMY32	100 genomes	A	92–123	Clinical	CA, USA	HMY559
HMY67	100 genomes	A	NRRL Y‐1546	Wine	West Africa	
HMY72	100 genomes	A (+)	Sigma1278b	Lab	Unknown	
HMY98	100 genomes	A	NRRL YB‐4506	Oak Tree	Japan	
HMY217	100 genomes	A (+)	CBS 7833	Clinical	MO, USA	HMY593
HMY47	100 genomes	B	96–100	Clinical	Italy	
HMY57	100 genomes	B	NRRL YB‐4348	Clinical	Portugal	HMY560
HMY169	Sanger	B	SK1	Soil/Lab	USA	
HMY268	Sanger	B	378604X	Clinical	Newcastle, UK	
HMY276	Sanger	C	UWOPS3461.4	Bertram Palm	Malaysia	
HMY171	Sanger	C	UWOPS05217.3	Bertram Palm	Malaysia	

*Note*: +, indicates a 15‐amino acid insertion in the A domain of *FLO11*.

A subset of strains was transformed to express a green fluorescent protein (GFP) under the control of the native *FLO11* promoter using a standard lithium acetate procedure (Gietz & Woods, 2002). Transformation cassettes were amplified with Phusion polymerase (New England BioLabs) and primers targeted to the *FLO11* open reading frame and *GFP‐kanMX* cassette (forward: 5′‐aacatcgtaatgaagaaacgaacatgttggaattgtatcATCGATGAATTCGAGCTCG; reverse: 5′‐tacttttgtaggcctcaaaaatccatatacgcacactATGAGTAAAGGAGAAGAACTTTT; lowercase letters indicate homology to yeast genome) using plasmid pFA6a‐*GFP*(S65T)‐*kanMX6* (Addgene plasmid #39292) as a template (Bähler et al., 1998). Transformants were selected on YPD agar supplemented with G418 (200 μg/ml) and verified via PCR and Sanger sequencing. Before transformation, a single spore isolate of HMY3 was isolated, HMY394.

### 2.2 Mat formation

Mats were inoculated with 5 μl of overnight YPD liquid cultures (1% yeast extract, 2% peptone, 2% dextrose) onto 1‐day‐old low agar YPD (0.3% agar) 60 x 10 mm Petri dishes. The plates were wrapped in parafilm and incubated at 25°C for 10 days with the mat surface facing upward. For each strain, four plates were inoculated: two for destructive sampling on Day 5 and 2 for destructive sampling on Day 10. The assay was repeated six times, for a total of 12 replicate mats at each time point to ensure reproducibility of results.

### 2.3 pH and glucose gradients

Five days after inoculation, two replicates of each strain were sampled in three locations: the area in the center of the mat (the hub), the area between the edge of the mat and the uncolonized agar (the rim), and the area furthest from the mat (the far rim) (Figure 1a). Samples were extracted as plugs using the wider side of a sterile 200 μl pipette tip and placed in 1.5 ml microcentrifuge tubes. The extracted agar was heated to 100°C for 2–5 min until liquified, then pipetted onto both pH and glucose strips (Diastix Reagent Strips for Urinalysis). For the first two replicate assays, broad‐range pH strips were used to determine the range found in the mats (Cytiva Whatman pH Indicator Papers, pH range 4.5–10 with 0.5 graduations). For the subsequent four assays, more precise strips with a narrower range were used (MilliporeSigma MColorpHast pH Test Strips and Indicator Papers, pH range 4–7 with 0.1 graduations). Only the data from the more precise measurements were used in the analysis. The sampling process was repeated for the remaining plates after ten days of incubation. All mats were imaged on an Olympus SZX16 dissecting scope before destructive sampling to verify similar phenotypes between the six independent assays and to measure mat size.

### 2.4 Mat size measurements

The images of the 5‐ and 10‐day mats were processed in Fiji (Schindelin et al., 2012) to determine the number of pixels representing mat growth (vs. background medium). Due to variability between assays, rather than using an absolute quantification of size, we summed all the pixels that represented growth in a given assay and calculated the percentage of growth represented by each strain. This allowed a direct comparison of the strains among assays.

### 2.5 Liquid boundary measurement

In four of the assays, 24 h after inoculation, the edge of one mat from each strain was imaged using a Zeiss Axioscope. The size of the liquid boundary was measured in pixels at four randomly chosen locations across the image using Fiji (Schindelin et al., 2012); pixel length was subsequently converted to distance in micrometers.

### 2.6 *prFLO11‐GFP* expression

Five of the original strains were transformed to replace one genomic copy of *FLO11* with *GFP‐kanMX*. For each strain, overnight cultures were imaged to determine starting *GFP* expression level, and two YPD mats were inoculated. The rim and hub of the mats were imaged every 12 h on a Zeiss Axioscope with LED fluorescence and a CMOS camera; image exposure and fluorescence gain were constant throughout imaging of all strains and timepoints. Each strain was monitored four times with independent cultures on different days to ensure the reproducibility of results. During two of these experiments, two low dextrose (0.1%) YPD mats were also inoculated and imaged using the same parameters. Images were subsequently processed in FIJI (Schindelin et al., 2012).

#### 2.6.1 Statistical analysis

The data were analyzed in JMP 11.2.0 using a generalized linear model (GLM). Glucose levels and pH were analyzed separately by fitting the following model to the data: *Y* = *α* + Strain*X_1_ + Day*X_2_ + Location*X_3_ + Assay*X_4_ + Strain_x_Location*X_1_ *X*_3_. The Day coefficient refers to the day the mats were sampled (5 or 10), Location refers to the sampling location (hub, rim, and far rim), and Assay refers to the independently performed assays.

## 3 RESULTS

**Figure 2:**
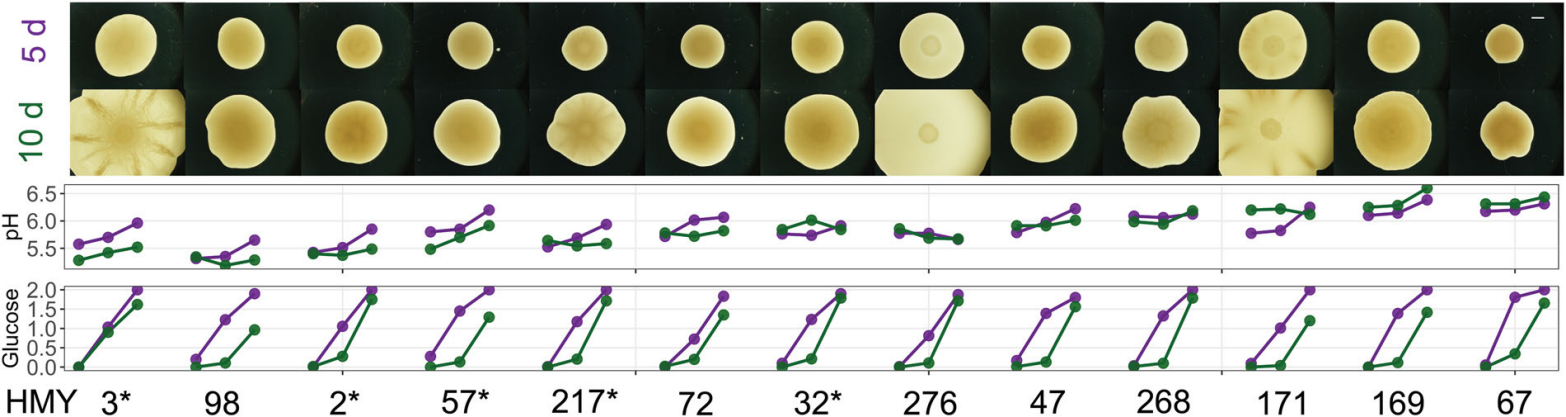
Mat diversity and associated gradients. Representative photos of the mat phenotype for each strain at 5‐ and 10‐days maturity; scale bar represents 2 mm. Glucose and pH gradients are plotted from hub to far rim for each strain using the average over 10 or 8 replicates (data as in Figure 1). Asterisks denote strains further investigated for *FLO11* expression

**Figure A1:**
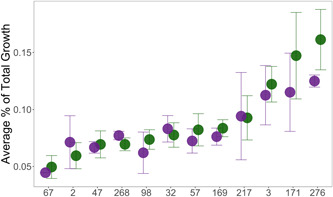
Mat area. The area of each mat in pixels was calculated as the percent of the area of all growth in all strains in a given assay

To test whether the characteristics associated with *S. cerevisiae* mat biofilm formation reported for strain Σ1278b were universal, we assayed the same characteristics in a panel of environmental yeast strains chosen to represent mat phenotypic diversity (Figure 2). The mats varied reproducibly in size (Figure A1, Appendix 1) and cabling complexity. Specifically, pH and glucose gradients were measured, along with the spatial patterning of *FLO11* expression. We hypothesized that the strength of these characteristics would vary with the strength and visual complexity of the mat phenotype.

### 3.1 Ph gradient

In the first two replicate assays, pH measurements over all strains, locations, and timepoints had a range of pH 4.5–7. The subsequent four replicate assays used more precise pH strips; these data are presented and analyzed here. The data were fitted to a GLM (Table 2); strain, assay, and location of the sample (hub, rim, and far rim) provided significant explanatory power to the model. Thus, it appears there was indeed a detectable pH gradient and significant variation among the pH environments generated by the strains, as well as between measurements in the replicated assays. Day of sampling (5 or 10) and the interaction between strain and location did not provide explanatory power to justify differences among strains in the strength of their gradients.

**Table 2:** pH generalized linear model results

Model	−Log likelihood	L‐R Chi‐square	*df*	*p*‐value
Difference	192.384	384.77	42	<0.0001
Full	265.973			
Reduced	458.357			
**Source**				
Assay		204.21	3	<0.0001
Strain		223.51	12	<0.0001
Location		25.52	2	<0.0001
Day		2.15	1	0.1423
Strain × Location		12.76	24	0.97

*Note*: Pearson goodness of fit: *χ* ^2^ = 84.72, *df* = 504, *p* = 1.00, suggesting the model fit the data reasonably well.

**Figure A2:**
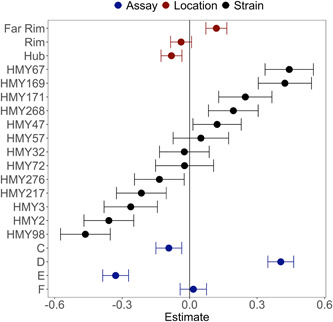
Coefficient estimates for the pH generalized linear model. The intercept for the equation was estimated to be 5.849

When all strains were combined, the average hub, rim, and far rim values at Day 5 were 5.76, 5.83, 6.04 and ranged from 5.31 to 6.18, 5.35 to 6.2, and 5.65 to 6.39, respectively. On average, there was a gradient from the inner hub to the outer rim, and finally to the agar at the far rim where there are no cells or growth. The estimates of the location coefficients support this interpretation (Figure A2, Appendix 1). At 10 days, the average pH values for the three locations were 5.79, 5.79, and 5.88, and ranged from 5.28 to 6.31, 5.19 to 6.31, and 5.29 to 6.6, respectively. These data suggest that the pH gradient within the mat itself began to weaken as the mats matured further. The high amount of variation in far rim pH measurements may be related to the different final sizes the mats achieved.

When strains were considered individually, there was variation among the strains in the measurements at the three locations (Figure 1). When the three locations were plotted together for each strain (Figure 2), the strain‐to‐strain variation in the overall pH environment was evident, as was an apparent lack of gradient in some strains (e.g., HMY276). However, the variation in gradients (the slopes of the gradients) was not significant in our model. The pattern of a weakening gradient can be detected. In numerous strains, the green 10‐day line appears flatter and lacks a slope in comparison to the purple 5‐day line. For some strains, this may be because the mat itself reached the edge of the plate (i.e., HMY171), but this is not true for all strains.

The variation in pH environment among the strains appeared to remain consistent over time, with the strains generating lower pH environments remaining low in comparison to strains generating higher pH environments (e.g., HMY98 vs. HMY169). The estimates of the coefficients for the strains suggest that overall HMY47, HMY67, HMY169, HMY171, HMY268 increase the pH compared to average, while HMY276, HMY217, HMY3, HMY2, and HMY98 decrease the pH compared to average, as the confidence limits do not encompass 0 (Figure A2, Appendix 1). Notably, the size and complexity of the mat did not appear to be associated with the pH environment. The three strongest mat biofilm‐formers, HMY3, HMY276, and HMY171, were scattered throughout the range of pH environments.

### 3.2 Glucose gradient

**Figure A3:**
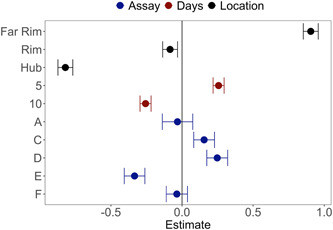
Coefficient estimates for the glucose generalized linear model. The intercept for the equation was estimated to be 0.823

**Figure A4:**
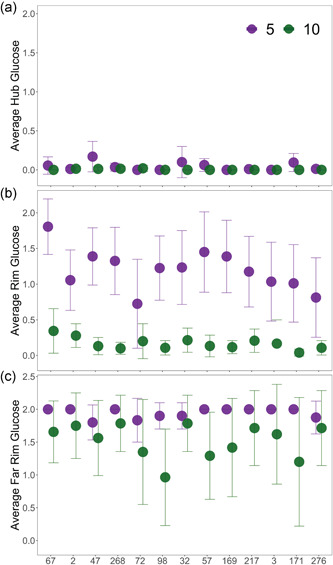
Glucose measurements. (a–c) Hub, rim, and far rim glucose measurements (±2 S.E.M.) in the panel of strains averaged over 10 replicates (two mats in each of five assays). Data are presented in order of smallest to lowest mat size at 10 days (as in Figure A1); 5‐day measurements in green and 10‐day measurements in purple

Glucose measurements were taken in five of the independent assays. The data were fitted to a GLM (Table 3); day of sampling (5 or 10), location of the sample (hub, rim, and far rim), and assay provided significant explanatory power to the model, while strain and the interaction between strain and location did not. Thus, it appears that there was a universal glucose gradient in all strains that changed over time, as well as variation in measurements between the replicated assays. Overall, the hub contained very little glucose, while at the rim, there was still some available, and at the far rim, the glucose concentration remained high (Figure 2). The estimates of the coefficients for location support this interpretation (Figure A3, Appendix 1). Previous research demonstrated that the availability of glucose at the rim increased with mat complexity (Regenberg et al., 2016). The same pattern is not apparent in our data (Figure A4b, Appendix 1).

**Table 3:** Glucose generalized linear model results

Model	−Log likelihood	L‐R Chi‐square	*df*	*p*‐value
Difference	412.21	824.42	43	<0.0001
Full	387.32			
Reduced	799.53			
**Source**				
Assay		114.08	4	<0.0001
Strain		12.91	12	0.3758
Location		725.32	2	<0.0001
Day		148.93	1	<0.0001
Strain × Location		24.89	24	0.4119

*Note*: Pearson goodness of fit: *χ* ^2^ = 125.57, *df* = 559, *p* = 1.00, suggesting the model fit the data reasonably well.

On Day 5, all strains had very similar glucose gradients, and the average measurements of the hub, rim, and far rim, were 0.074%, 1.2%, and 1.95%, with the strain averages ranging from 0% to 0.29%, 0.725% to 1.47%, and 1.83% to 2%, respectively. At this time, the rim still had significant glucose available and was the most variable of the locations, while the far rim had close to the concentration of the YPD medium before inoculation (Figure A4, Appendix 1). At Day 10, the average measurements at the hub, rim, and far rim were 0.005%, 0.22%, and 1.52%, with the strain averages ranging from 0% to 0.2%, 0.04% to 0.34%, and 0.96% to 1.79%, respectively. At this time, there was very little glucose left at the rim and the far rim was the most variable of the locations, with some of its glucose being consumed by the mats of differing sizes.

### 3.3 Liquid boundary

**Figure 3:**
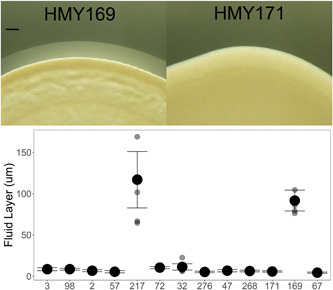
Liquid layer surrounding mats. Liquid surrounding mats 24 h after inoculation, averaged over four replicates (±2 S.E.M.). Representative photos showing the layer ~48 h after inoculation; scale bar represents 200 μm

**Figure A5:**
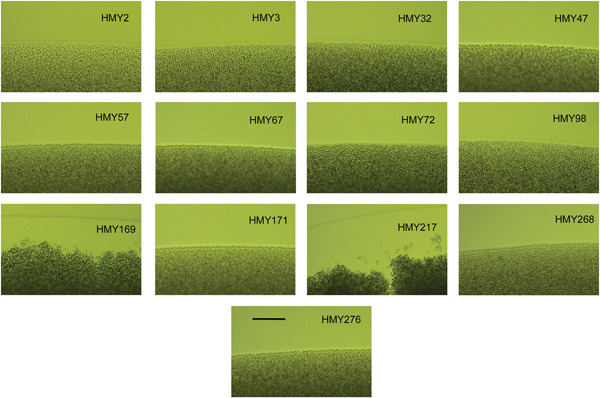
Liquid layer surrounding mats in all strains. Representative photos used to measure the liquid layer surrounding mats. The photos were taken 24 h after mat inoculation; the length of the liquid layer was measured in the number of pixels and subsequently converted into micrometers. Scale bar represents 100 μm

*Saccharomyces cerevisiae* mats are surrounded by a liquid layer containing shed mucins. The size of the liquid layer was measured after 24 h of growth (Figure 3). All mats had visible and measurable liquid boundaries (Figure A5, Appendix 1); however, surprisingly, only two had voluminous and extensive liquid surrounding the mats. The genetic backgrounds that had the larger liquid layer were consistent among replicates. Strains that were transformed to contain *GFP‐kanMX* in place of one copy of *FLO11*, and were thus hemizygous for *FLO11*, also produced a liquid boundary layer. HMY593 (derived from HMY217) produced a robust layer (although smaller than when the diploid at *FLO11*) while the others did not, consistent with the original isolates (Figure A10, Appendix 2). Also surprisingly, the size and complexity of the mature mats did not appear to be associated with having a robust liquid layer. Instead, the two strains producing the most liquid were medium‐sized mats with some detectable complexity (cables and rough edges).

### 3.4 *prFLO11‐GFP* expression

**Figure 4:**
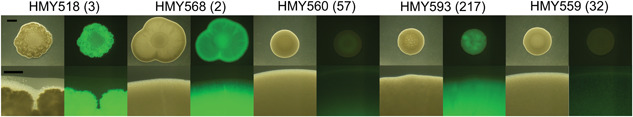
Representative mat biofilms with *prFLO11‐GFP* strains. The top row scale bar represents 3 mm; the bottom row scale bar represents 500 μm. All images were captured and processed the same way to highlight the differences among expression levels

Five strains were engineered to replace one copy of *FLO11* with *GFP*; *GFP* expression was therefore under the control of the *FLO11* promoter and used as a proxy for the initiation of *FLO11* expression. GFP is very stable in yeast, so its presence indicates that *FLO11* has been expressed in the cell, but does not necessarily indicate continuous expression. GFP was monitored at the rim and hub every 12 h during the beginning of mat formation. Image acquisition and processing were standardized across all strains and timepoints allowing comparison of the spatial arrangement of GFP expression (Figure 4) and expression level among strains over time. However, due to the natural variation in mat structure among the strains, the images were not quantitatively analyzed, as the thickness of the mats might influence the results.

**Figure A6:**
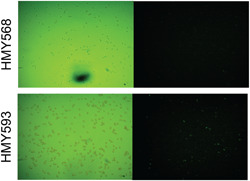
*prFLO11‐green fluorescent protein* expression in overnight liquid cultures. Representative images showing variegated expression in two different strains

Overnight cultures were imaged on a hemocytometer to determine the proportion of cells expressing GFP when the mats were inoculated (Figure A6, Appendix 1, 2). Strains HMY559 and HMY560 had no detectable GFP, HMY518, and HMY568 had ~0.5%–1% of cells strongly expressing GFP, and HMY593 had ~2% of cells strongly expressing GFP, but a further subpopulation with lower levels of GFP (~15%).

**Figure A7:**
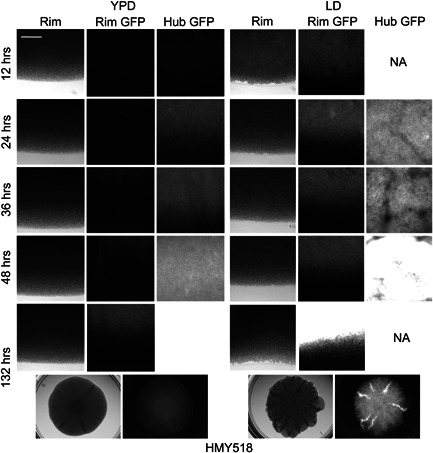
Green fluorescent protein time‐lapse imaging for strain HMY518. Scale bar represents 200 μm

**Figure A8:**
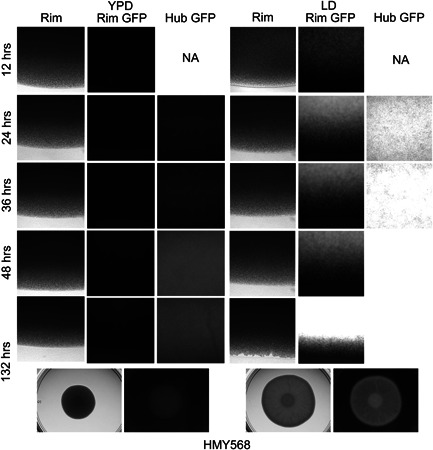
Green fluorescent protein time‐lapse imaging for strain HMY568

**Figure A9:**
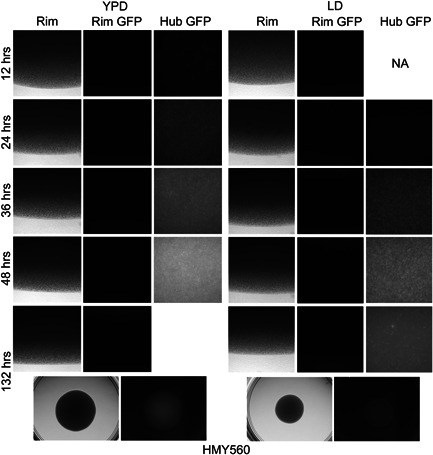
Green fluorescent protein time‐lapse imaging for strain HMY560

**Figure A10:**
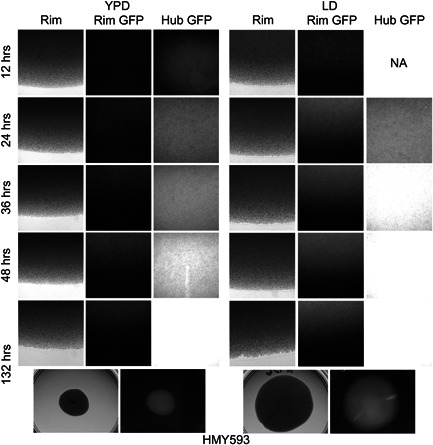
Green fluorescent protein time‐lapse imaging for strain HMY593

**Figure A11:**
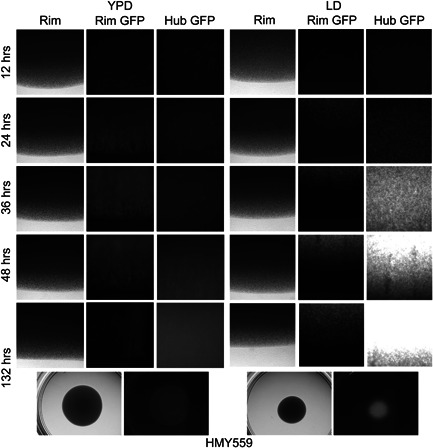
Green fluorescent protein time‐lapse imaging for strain HMY559

As mat formation began, GFP was detectable as early as 12 h in some strains; by 24 h, it was detectable in the hub of all strains. By 48 h, the characteristic spatial arrangement of expression of the strain was apparent. As *FLO11* expression is tightly linked to glucose availability (Kuchin et al., 2002), mat formation was also monitored on low dextrose YPD (LD), which decreased glucose from 2% to 0.1%, a level similar to that found in the hub of mature mats formed on YPD. In most cases, this led to earlier and increased *GFP* expression, but not a change in the spatial arrangement (Figures A6, A7, A8, A9, A10, A11, Appendix 2). In general, GFP was visible in all mats within 12 h of inoculation on LD. The spatial characteristics are briefly described here, in the order of the pH environment of the strains, from lowest to highest.

HMY518 (Figure A7): On 2% glucose, seemingly variegated GFP was detectable in the hub at 24 h, but became more uniformly detectable and at a significantly higher level by 48 h. Throughout monitoring, there was very little GFP at the rim, although variegated GFP was detectable moving inward toward the hub. In comparison, on 0.1% glucose, GFP was detectable all the way out to the rim as early as 12 h after inoculation. The mat itself was more ruffled and cabled and had a much higher GFP signal overall. In both cases, the cells at the very edge of the expanding rim never produced a GFP signal, while the rest of the mat had bright GFP cells throughout.

HMY568 (Figure A8): On 2% glucose, the first appearance of GFP was a small amount of variegated signal at 24 h in the hub. The signal in the hub slowly increased through 48 h, but never to a high level of expression. Throughout monitoring, no GFP was detectable in the rim. In comparison, on 0.1% glucose, the variegated GFP signal was detectable all the way out to the rim as early as 12 h after inoculation. The mat itself spread out further and with more structural complexity. The hub had very high levels of signal in all the cells as early as 24 h and maintained variegated expression at the rim. In both cases, the cells at the very edge of the expanding rim produced no or less GFP signal than the hub, which had uniform expression.

HMY560 (Figure A9): On 2% glucose, the first appearance of GFP was a small amount of variegated signal at 24 h in the hub. The signal in the hub increased through 48 h but was never detectable in the rim. In contrast to other strains, on 0.1% glucose, there was less GFP signal overall. The mat itself was smaller, likely due to less available glucose. In both cases, there was GFP in the center and none in the outer rim; in comparison to other strains, this strain had significantly less GFP signal.

HMY593 (Figure A10): On 2% glucose, significant GFP was detectable in the hub at 12 h, and variegated expression toward the rim by 24 h. By 48 h, there was a strong signal in the hub and less toward the rim. In comparison, on 0.1% glucose, GFP was detectable toward the rim as early as 12 h after inoculation. The mat itself was more ruffled and cabled and had a much higher GFP signal overall. In both cases, the cells at the very edge of the expanding rim did not produce a GFP signal, while the rest of the mat had bright GFP throughout. This strain appeared to have the strongest signal of all the strains.

HMY559 (Figure A11): On 2% glucose, unlike the other strains, the first appearance of GFP was a variegated signal at the outer edge of the rim (rather than the hub) at 12 h. The signal at the rim remained variegated but became stronger through 48 h. The signal in the hub was weaker but increased over time. In comparison, on 0.1% glucose, the mat was smaller, and as with other strains, the GFP signal was greater overall. The variegated signal first appeared in the hub at 12 h, then became stronger in both the rim and the hub through 48 h. The spatial pattern changed from variegated GFP throughout the whole mat in 2% glucose to no GFP in the expanding outer edge in 0.1% glucose.

Overall, there was variation in the amount of GFP detected in the different strains, the time and location that GFP was first detected, and whether the signal was variegated or seemingly more uniform. However, certain characteristics seemed to be universal: (1) GFP was detected early in mat formation, between 12 and 24 h of inoculation, (2) a decrease in glucose concentration led to an increase in GFP signal (except for HMY560), (3) at the very edge of the expanding rim of the mat, there was no GFP signal (except for HMY559 on 2% glucose), and (4) the hub had the strongest GFP signal.

## 4 DISCUSSION

The *S. cerevisiae* mat represents a potentially ecologically significant, multicellular phenotype that can be studied and manipulated in the lab, and that has mostly been characterized in a single genetic background. We took advantage of the availability of environmental *S. cerevisiae* isolates, which have been collected globally in a variety of ecological niches (Liti et al., 2009; Strope et al., 2015), to determine whether there is a suite of universal mat characteristics. We hypothesized that the traits would vary in relation to mat cabling/complexity and size.

The strains used in this study represent a range of colony phenotypes observed on low agar medium (Hope and Dunham, 2014; Oppler et al., 2019; Regenberg et al., 2016). Unlike when studying a single genetic background in which biofilm and “smooth” mats can be easily differentiated, the phenotype is continuous in environmental isolates. The variability in the phenotype can also extend to replicates within a genetic background. In our experience, the overall appearance of replicate mats was qualitatively similar, but the absolute size, the amount of cabling, and other visual phenotypic characteristics often varied, despite attempts to be systematic in all experiments. This variability could be because of natural stochasticity in mat formation or because mat formation is sensitive to small fluctuations in environmental conditions (i.e., small differences between batches of medium), or both. These factors may explain differences detected among our replicate experiments. Despite the variability in the phenotype and among assays, certain mat characteristics were universal, while others were consistently different among the environmental isolates.

The first characteristic we investigated, the existence of a pH gradient across the mat and medium, showed significant variation. More specifically, we found evidence for overall differences in the pH environment generated by the isolates, but not necessarily differences in the strength of the gradient (i.e., a steeper slope in some strains vs. others). Surprisingly, the isolates with the most dramatic mats (based on size and complexity) did not have the strongest gradients or the lowest pH environments. It is worth noting that pH may be of particular ecological relevance to budding yeast. The adherence properties of the adhesin Flo11, which is required for adherence to surfaces and other cells, vary with pH (Kraushaar et al., 2015). Interestingly, so does the effectiveness of the killer toxin, which is most lethal in acidic conditions (Lukša et al., 2016). The killer toxin is a warfare phenotype (Boynton, 2019) detected in up to 10% of environmental isolates (Pieczynska et al., 2013), in which killer yeast secrete a protein that kills nearby sensitive cells, but not other toxin‐producers (Schmitt & Breinig, 2006). The killer and biofilm phenotypes may interact with one another (Deschaine et al., 2018) to structure natural communities, and pH may be a mediating factor. Thus, it is of particular interest that the pH environments generated by the isolates studied here vary significantly.

Next, we investigated the existence of a glucose gradient. While it is not surprising that there would be a gradient from the center of the mat out to the uncolonized medium, the glucose level at the expanding front was of particular interest. We hypothesized that glucose at the rim would correlate with the size of the mat, as the ability to quickly spread across viscous surfaces may be associated with not immediately utilizing all available glucose (Regenberg et al., 2016). Regenberg et al. (2016) found more glucose at the rim of biofilm mats than nonbiofilm mats, supporting their hypothesis. While we detected a glucose gradient in all strains, which changed over time, we found no evidence that the gradient varied among isolates. Most importantly, the glucose level at the rim did not appear to vary with the size of the mat. There was, however, some minor variation in rim glucose at 5 days and in far rim glucose at 10 days. It is possible that our glucose assay was not sensitive enough to detect biologically significant differences among the strains.

The third mat characteristic we investigated was the size of the liquid layer surrounding the mats, which is hypothesized to act as a lubricant, as well as function in the transport of nutrients and signaling molecules (Karunanithi et al., 2010; Pitoniak et al., 2009). Unexpectedly, most strains produced a small liquid layer. Only two strains had a pronounced layer, neither of which generated large, complex mats. Both strains produced medium‐sized mats with some evidence of cabling, and each had a different pH environment. Thus, despite our hypothesis that the liquid layer was crucial to mat biofilm formation, our data suggest otherwise.

Finally, after investigating the physical mat characteristics of the entire panel, we chose a subset of strains to characterize the timing and location of the initiation of *FLO11* expression during mat formation on rich and low dextrose media. In these strains, *GFP* was integrated into the genome and under the control of the endogenous complex *FLO11* promoter. There was no degron and GFP is highly stable in yeast (Mateus & Avery, 2000), so the appearance of GFP simply indicated that the cell had at some point expressed *FLO11*. Nonetheless, differences in the amount of GFP and the location it was detected differed between the strains and media. A few observations from this investigation are worth noting.

First, comparing growth on a rich medium with growth on a low glucose medium proved to be a test for the ability of a strain to form a biofilm. When there was a decrease in the amount of glucose in the medium, three of the strains generated larger, more cabled mats with more detectable GFP (HMY518, HMY568, and HMY593), supporting previous observations of the effect of glucose concentration (Reynolds et al., 2008). This suggests that these are biofilm‐forming strains that increase *FLO11* expression and surface spreading in low glucose conditions, despite the fact that not all of them initially formed the characteristic large, floral pattern when grown on YPD (i.e., HMY2 and HMY217). The other two strains (HMY559 and HMY560) formed smaller, smooth mats when there was a decrease in the amount of glucose, which is what would be expected if the strains were not biofilm‐formers (i.e., less carbon source usually means smaller colonies and lower biomass). Interestingly, one of these nonbiofilm‐forming strains, HMY559, did show an increase in detectable GFP when grown in low glucose conditions. This suggests that more *FLO11* was expressed, despite the absence of a biofilm; the low glucose signal functioned as expected, but the effect of *FLO11* expression was different. It is possible that genetic variation elsewhere in the genome was preventing the strain from forming a biofilm. The other nonbiofilm‐forming strain, HMY560, had less detectable GFP in the low glucose conditions, and presumably less *FLO11* expression, which was the opposite of the other four strains.

Next, the larger and more complex a mat biofilm (i.e., the more similar it was to the characteristic phenotype), the more GFP signal was detected. This could be due to more cells expressing GFP, more GFP being expressed in individual cells or both. The relationship between GFP and mat complexity was true among the strains, as well as within a genetic background when glucose availability was altered. This observation is not surprising given the role Flo11 plays in mat biofilms; however, by investigating expression in environmental isolates, we confirm its central role in mat biofilm formation in multiple genetic backgrounds and conditions. Interestingly, as noted above, significant expression was also detected in nonbiofilm‐forming strains.

Related to this observation, in the biofilm mats, the hub consistently had the strongest GFP signal, while the expanding rim had nearly none. This observation may be simply due to a difference in time: the longer the cells have been in the biofilm, the more likely they are to have expressed *FLO11* at some point. Another possibility is differences in spatial expression patterns due to the microenvironment. The lower pH and absence of glucose in the center of the mats may have led to increased expression. In the nonbiofilm mats, the GFP signal was still detected, although it was weaker overall, and it generally (but not always) conformed to the same spatial arrangement: strongest in the hub and weak or nondetectable at the edge of the expanding rim. Previous research on the Σ1278b background using qPCR found consistent *FLO11* expression throughout the entire mat (Reynolds et al., 2008). The discrepancy between the two sets of results could be due to differences in the genetic backgrounds or the sensitivity of the different approaches. The absence of GFP at the rim was only at the very expanding edge of the biofilm mat (Figures A6, A7, A8, A9, A10, A11); mats sampled for qPCR would likely include sections further into the rim. Furthermore, qPCR detects ongoing expression, while our approach detects that *FLO11* expression was initiated during growth and does not necessarily imply continued expression.

When considering all the characteristics presumed to be associated with mat biofilms: a pH gradient and lower overall pH environment, available glucose at the rim, a robust liquid layer, and high levels of *FLO11* expression, the most “biofilm” strain in our panel was HMY217 (originally, YJM128, or CBS 7833). However, simple visual inspection of the mat phenotype would have identified HMY3 (YJM311), HMY171 (UWOPS 05 217.3), and HMY276 (UWOPS3461.4) as the most extreme versions of the mat biofilm phenotype (Figure 2). It is worth noting that none of the traits we investigated, including size and complexity, seemed to be associated with a particular *FLO11* regulatory allele (Oppler et al., 2019).

Our results beg the question of what defines a mat biofilm. Biofilms are generally understood to be cooperative, differentiated microbial communities that are structured by an extracellular matrix and anchored to a surface, and that have increased resistance to environmental stressors (Blankenship & Mitchell, 2006; O'Toole et al., 2000). In the case of *S. cerevisiae*, we would argue that visual morphological complexity is an indicator of cellular differentiation and cell–cell attachment, and therefore is a good indicator of biofilm formation, especially when such morphological complexity increases in response to nutrient scarcity. However, it is unclear which characteristics that were once presumed to be associated with mat biofilms are indicative of biofilm formation, as opposed to simple growth on low agar medium. It is possible that the formation of glucose and pH gradients would occur, regardless of whether there was the type of differentiation associated with biofilms. The traits investigated here vary continuously and independently of one another, including the ability to spread widely over the surface of semi‐solid agar. Rather than classifying isolates as biofilm‐formers and nonbiofilm‐formers, an alternative would be to consider multiple properties of the communities generated by these isolates. As with most natural phenotypes, environmental isolates of yeast will most likely exist on a continuum of noncooperative, nondifferentiated piles of cells to highly structured, cooperative communities.

Further research into this phenotype may provide insight into how environmental conditions may select different sets of traits to adapt this model organism to its myriad ecological niches. Although this study focused on a small panel of strains and demonstrated unexpected variation in certain traits, the availability of isolates collected globally from a wide variety of habitats (Peter et al., 2018) will allow this phenotype to be investigated on a large scale. For example, associating mat traits, like the pH environment generated by a strain, with the niche from which it was collected may provide insights into which traits are favored under different ecological circumstances. Furthermore, uncovering natural genetic variants that control mat biofilm formation, as has been done for other complex life‐history traits in yeast (De Chiara et al., 2022), or further investigating the biochemical properties of natural *FLO11* variants (Oppler et al., 2019) and the conditions under which they generate the greatest cell‐cell adhesion (Bouyx et al., 2021; Brückner et al., 2020) could provide insight into the molecular basis of how multicellularity is favored in this organism.

## AUTHOR CONTRIBUTIONS

Amy L. Forehand: Data curation (lead); investigation (lead); writing—review & editing (supporting). Dulguun Myagmarsuren: Data curation (equal); investigation (lead); methodology (lead); writing—review & editing (supporting). Ziyan Chen: Data curation (equal); investigation (equal); writing—review & editing (supporting). Helen Murphy: Conceptualization (lead); data curation (equal); formal analysis (lead); funding acquisition (lead); project administration (lead); writing—original draft (lead); writing—review & editing (equal).

## CONFLICT OF INTEREST

None declared.

## ETHICS STATEMENT

None required.

## Data Availability

Strains generated for this study are available upon request from the corresponding author. The pH and glucose data set has been deposited at figshare: https://doi.org/10.6084/m9.figshare.19270688.v1

## APPENDIX 1

This appendix contains figures related to the analysis of the main mat biofilm phenotypes being investigated: pH, glucose concentration, and liquid boundary formation.

 Figures A1, A2, A3, A4, A5

## APPENDIX 2

This appendix contains images related to the analysis of *pr‐FLO11‐GFP* expression during mat formation in five focal strains.

Before inoculating mats, the liquid cultures were imaged (e.g., Figure A6). Mat formation was then documented over 5 days. Image acquisition and processing were standardized across all strains and timepoints. In Figures A7, A8, A9, A10, A11, the first three columns represent mat formation on low agar YPD, while the next three columns represent mat formation on low agar low dextrose YPD (LD). For both types of media, the first column is a brightfield image of the edge of the colony, while the second column is the corresponding GFP image. The final column is a GFP image of the hub of the mat. No brightfield is available, as the mats are dense and cannot be penetrated by the light. The rows represent different time points. At the final time point, 132 h, there are also brightfield and GFP images of the entire mat.
